# Damage of testicular cell macromolecules and reproductive capacity of male rats following co-administration of ethambutol, rifampicin, isoniazid and pyrazinamide

**DOI:** 10.2478/v10102-012-0002-9

**Published:** 2012-03

**Authors:** Ganna Mykhailivna Shayakhmetova, Larysa Borysivna Bondarenko, Valentina Mykolaivna Kovalenko

**Affiliations:** Institute of Pharmacology & Toxicology, National Academy of Medical Sciences of Ukraine, Eugene Potier St. 14, Kyiv, 03680, Ukraine

**Keywords:** antituberculosis drugs, male reproductive capacity, DNA-fragmentation, testis, rat

## Abstract

The necessity to minimize adverse effects of tuberculosis chemotherapy requires a comprehensive evaluation of the effects of antituberculosis drugs on the reproductive system and testicular cell macromolecules. The epidemiological situation of tuberculosis in Central and Eastern Europe is getting worse. Data on adverse effects of antituberculosis drugs are scare concerning particularly their effects on the reproductive system. The aim of the present study was to investigate the potential effect of ethambutol, rifampicin, isoniazid and pyrazinamide co-administration on lipid peroxidation, glutathione content and protein SH-groups, DNA fragmentation levels, the reproductive capacity of Wistar male rats and the antenatal development of their posterity. The rats (150–170 g) were divided into two groups: group I – received antituberculosis drugs suspended in 1% starch gel per os: ethambutol – 155 mg/kg b.w./day, rifampicin – 74.4 mg/kg b.w./day, isoniazid – 62 mg/kg b.w./day, pyrazinamide – 217 mg/kg b.w./day, group II (control) – received only starch gel in corresponding volumes. The contents of TBA-active compounds, glutathione and protein SH-groups in testis and sperm were determined spectrophotometrically, the DNA-fragmentation was determined using an UV transilluminator (BIORAD, USA), reproductive system indices were measured by standard methods. The co-administration of therapeutic doses of ethambutol, isoniazid, rifampicin and pyrazinamide to male rats during the period of spermatogenesis caused an increase in the rate of thiobarbituric acid reactive substances formation in testis and sperm, decrease of testis glutathione and protein SH-group contents, significant changes in DNA fragmentation, fatal decrease of male fertilizing capacity and fertility, and increase of pre- and post-implantation embryo lethality. The changes in reproductive indices could be the result of direct or indirect effects of one or more drugs investigated.

## Introduction

In 2004, the WHO Assembly announced protection of reproductive health as a world-wide priority and approved the first international strategy on this problem (World Health Organization [webpage in the internet]). Of the potential risks for reproductive health, special concern was given to the ability of a great number of xenobiotics (including medicines) to affect the function of the male reproductive system (Ten *et al.*, 2008).

A complex analysis of potential effects of chemicals on the reproductive system is urgently required for the development of improved treatment strategies and the formulation of shorter, more effective and safe regimens for the prevention and treatment of chronic diseases, at simultaneously minimizing adverse effects.

One third of the world's population has positive tuberculin tests and this number is increasing. Thus analysis of the potential effects of medicines on the reproductive system is important for improving first-line antituberculosis therapy as current therapeutic regimens are associated with a great number of adverse effects and can lead to potential risks for reproductive health. Understanding the nature and the severity of these adverse effects is very important. At present it is known that simultaneous and long-term use of antituberculosis drugs may cause various negative effects on the metabolism of amino acids and proteins and the rate of protein biosynthesis (Kovalenko *et al.*, 2007; Bondarenko *et al.*, 2006, Bondarenko *et al.*, 2011). This allows to suspect a potential negative effect on reproductive function.

The epidemiological situation of tuberculosis in Central and Eastern Europe keeps worsening (Arinaminpathy *et al.*, 2010). The data on adverse effects of antituberculosis drugs, particularly their effects on the reproductive system, are limited. The aim of the present study was to investigate the potential effect of ethambutol, rifampicin, isoniazid and pyrazinamide co-administration on male rat testis lipid peroxidation, glutathione contents and protein SH-groups, DNA fragmentation levels, reproductive capacity and on antenatal development of their posterity.

## Material and methods

Ethambutol, isoniazid, pyrazinamide and rifampicin were supplied by the SIC “Borzhagovsky Chemical-Pharmaceutical Plant” CJSC, Ukraine.

Wistar albino male (n=24) and female (n=48) rats, body weight (b.w.) 150–170 g (4 month old) were purchased from Biomodel Service (Kyiv, Ukraine). The animals were kept at standard conditions of nutrition, water and light regimens.

The study was carried out according to the national and international guidelines and the law on animal protection was observed. All animal studies were performed in accordance with the recommendations of the European Convention for the Protection of Vertebrate Animals used for Experimental and other Scientific Purposes and approved by the Institutional Animal Care and Use Committee.

The rats were kept for acclimatization during 10 days, then they were randomized into experimental and control groups. Each group included 12 males. Antituberculosis drugs suspended in 1% starch gel were given by gavage in DOTS (directly observed treatment, short-course) regimen at maximal doses used in clinic (Donate, 2006): ethambutol – 155 mg/kg b.w./day, rifampicin – 74.4 mg/kg b.w./day, isoniazid – 62 mg/kg b.w./day, pyrazinamide – 217 mg/kg b.w./day for 60 days (duration of spermatogenesis process and time of germ cell maturation in epididymis). The coefficient for conversion of human doses to animal equivalent doses based on body surface area was taken into account (Food and Drug Administration [webpage in the internet]). The control group received only starch gel in corresponding volumes (5 ml/kg b.w.). After 46 days of repeated administrations, the males from both groups were mated with intact females at the ratio 1 male: 2 females during 14 days (approximately 2–3 estrous cycles). During this period the administration of antituberculosis drugs to male rats was continued.

Effects of antituberculosis drugs on male fertilizing capacity were determined by the index:

$$\frac{\mathrm{Number of pregnant females}}{\mathrm{Number of females mated with males})}\times 100$$

The males were sacrificed under mild ether anesthesia via decapitation after completion of the mating period, 24 hours after the last aministration of the drugs. Their testes and epididymides were used for biochemical assays.

The contents of reduced glutathione and proteins SH-groups in testis homogenates were determined with Ellman's reagent (Sedlak *et al.*, 1968), lipid peroxidation was investigated as the rate of ascorbate-induced thiobarbituric acid reactive substances (TBARS) formation (Stalnaya *et al.*, 1977), protein contents by Lowry's method (Lowry *et al.*, 1951).

The DNA from testes was isolated by a modified method from Current Protocols in Toxicology (Zhivotosky & Orrenius, 2001). The tissue was homogenized and digested in digestion buffer (100 mM NaCl; 10 mM Tris-HCl; 25 mM EDTA, pH 8; 0.5% SDS and freshly added 0.1 mg/mL proteinase K) (Sigma-Aldrich, Inc., USA)) (1:1.2 mg/ml) with shaking at 50°C for 15 h. RNA was degraded by incubation of the samples with 1–100mg/mL thermostable RNase H for 1.5 h at 37°C. DNA was extracted with an equal volume of phenol:chloroform:isoamyl alcohol (25:24:1) and centrifuged for 10 min at 1 700×*g*. Then the DNA was precipitated by adding 0.5 vol 7.5 M ammonium acetate and 2 vol 100% ethanol to the aqueous layer; samples were separated by centrifugation at 1 700 × *g* for 5 min, rinsed with 70% ethanol, and air-dried. Pellets were dissolved in TBE buffer (10 mM Tris-HCl and 1 mM EDTA, pH 8) and then fractionated through 2% agarose gels (50–60 V; 3.5h). After electrophoresis, gels were stained with ethidium bromide and visualized under a UV transilluminator (BIORAD, USA). Analysis of electrophoresis data was carried out with Quantity One Software (USA).

The females were sacrificed under mild ether anesthesia via decapitation on day 20 of pregnancy for determination of fetal antenatal development indices. The number of corpora lutea in ovaries, of implantation sites and of live and dead fetuses in each uterine horn were counted after laparotomy of pregnant females. Indices of embryonic death at pre- and postimplantation periods of development were calculated according to standard procedures (Clegg *et al.*, 2001; Tyl, 2005).

The obtained data were calculated by one-way analysis of variance (ANOVA) and compared using the Tukey test. Differences were considered statistically significant at *p<*0.05.

## Results

In our experiments we demonstrated an increase of TBARS formation in rat testis (+15%) and epididymal suspension of spermatozoids (+38%) in the group with antituberculosis drugs co-administration in comparison with the control group (Table 1).

**Table 1:** The rate of ascorbate induced formation of TBARS in male rats testis homogenate and epididymal suspension of spermatozoids, nmoles/min × mg of protein (M ± S.E.M., n=12).

	Tissue	
Group	testis	epididymal suspension of spermatozoids
Antituberculosis drugs	0.302±0.031*	0.162±0.006*
Control	0.259±0.02	0.100±0.006

M ± S.E.M. – mean ± standard error of the mean

* *p*<0.05 statistically significant in comparison with control

The combined administration of antituberculosis drugs to male rats during the whole period of spermatogenesis caused a decrease of testis glutathione contents by 19% in comparison with control (Table 2). Simultaneously the content of protein SH-groups decreased in the testis by 22%.

**Table 2:** Contents of rat testis glutathione and protein SH-groups with combined administration of antituberculosis drugs, nmoles/mg of protein (M ± S.E.M., n =12).

Group	Contents of glutathione	Contents of protein SH-groups nmoles/mg of protein
Antituberculosis drugs	21.25±1.52 *	45.44±4.76 *
Control	26.32±0.91	58.41±3.64

M ± S.E.M. – mean ± standard error of the mean

* *p*<0.05 statistically significant in comparison with control

The DNA is an important molecular target for antituberculosis drugs. They can induce DNA lethal restriction by endonucleases (Burchiel *et al.*, 1992) and inhibit processes of DNA and RNA reparation by nucleus DNA-polymerases. The rate and character of DNA-fragmentation is a marker of apoptotic processes in the organism (Wang *et al.*, 2005).

**Figure 1:**
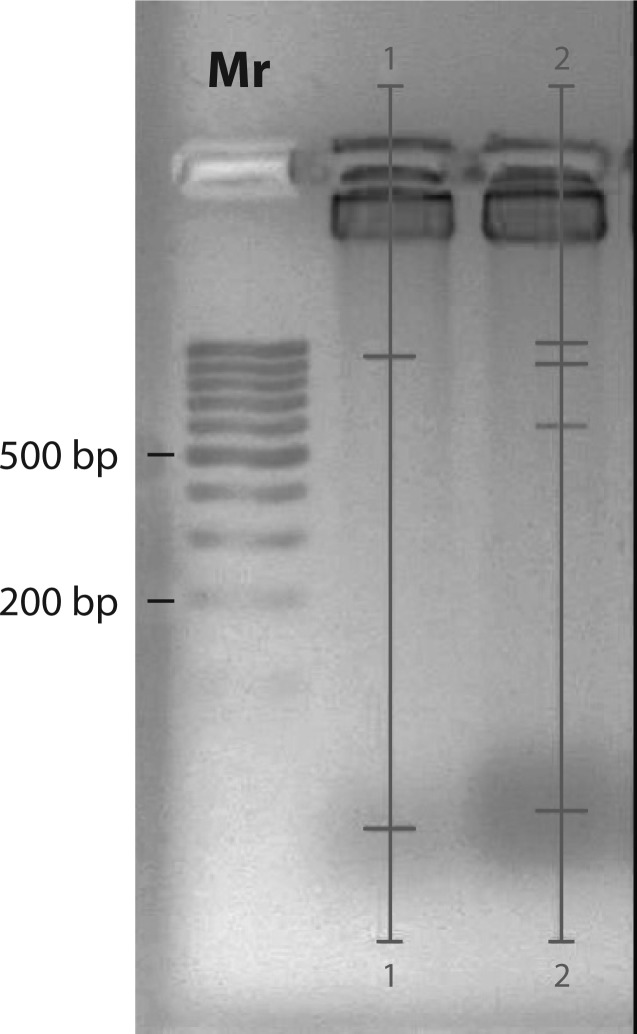
Levels of DNA fragmentation in rat testis (Mr – marker; lane 1 – control group; lane 2 – antituberculosis drugs co-administration). Analysis was carried out using the Quantity One Software.

In the present study, toxic damage of testis cells by co-administered antituberculosis drugs (confirmed by morphological data (unpublished observations)) was accompanied by changes of nuclear DNA fragmentation (Figure 1).

Co-administration of antituberculosis drugs caused DNA fragmentation in rat testis cells with four fractions of fragments in diapasons 1000 b.p., 900 b.p., 600 b.p. and lower molecular weight fraction with DNA fragments 40–30 b.p. Only two fractions of DNA fragments: main-30–20 b.p. and minor-1 000 b.p. were detected in the control group.

In our experiments the mating behavior of male rats (after 46 days of antituberculosis treatment) remained unchanged. Yet the smaller number of pregnant females in the experimental group could be evidence of decreased male fertility after antituberculosis treatment (Table 3). Of 24 mating females only 4 became pregnant, while the index of fertility in the control group was 91.7%.

**Table 3:** Rat male fertility index with combined administration of antituberculosis drugs.

Group of males	Number of mated females	Number of pregnant females	Fertility index, %
Antituberculosis drugs	24	4	17
Control	24	22	91.7

The number of living fetuses per one female was also lowered in comparison with the control group (Table 4). The comparison of embriolethality levels in experimental and control groups at different terms of embryonal development demonstrated great negative effects of antituberculosis drugs on male reproductive function. In the experimental group the levels of paternal mediated pre- and postimplantational lethality were 65.3% and 79.9%, respectively.

**Table 4:** Fertility and embryogenesis parameters on day 20 of gestation – day of mating intact rat females with male rats treated by combined administration antituberculosis drugs.

Male group indices	Control	Antituberculosis drugs
Number of pregnant females	22	4
Total number of corpora lutea	244	75
Number of corpora lutea per one female, M ± S.E.M.	11.09±0.37	18.75±3.59
Total number of implantation sites	219	26
Number of implantation sites per one female, M ± S.E.M.	9.95±0.59	6.50±3.33
Preimplantational loss, abs/% a	18 / 10.25	48 / 65.3
Preimplantational loss per one female, M ± S.E.M.	0.82±0.32	12.0±3.89*
Postimplantational loss, abs/% b	9 / 4.1	20 / 76.9
Postimplantational loss per one female, M ± S.E.M.	0.41±0.15	5.0±3.72
Total number of living fetuses, abs/%	217 / 99.09	7 / 27
Number of living fetuses per one female, M ± S.E.M.	9.08±0.52	1.75±1.43*

M ± S.E.M. – mean ± standard error of the mean

* *p<*0.05 statistically significant in comparison with control

a Preimplantational loss was calculated as: *% preimplantational loss = [(number of corpora lutea – number of implantation sites) / number of corpora lutea] × 100*

b Postimplantational loss was calculated as: *% postimplantational loss = (number of lost fetuses / number of implantation sites) × 100*

## Discussion

Antituberculosis drugs co-administration caused the production of oxygen active forms, activation of lipid peroxidation and oxidative stress development (Tasduq *et al.*, 2005). Oxidative stress, in turn, can damage all intracellular macromolecules (glutathione, DNA, RNA, proteins, lipids and ATP). Any changes in the level of these substances are of key importance for cell viability and great deviations cause cell damage and death (Cooke *et al.*, 2003; Jones, 2008).

On the one hand reactive oxygen species (ROS) play a central role for sperm physiology, such as sperm maturation and capacity. On the other hand, abnormal ROS production is associated with defective sperm function. The delicate balance between ROS production and recycling is essential for spermatogenesis. Excessive generation of seminal ROS can cause male infertility (Hsien *et al.*, 2006). Moreover, according to data of some authors (Minamiyama *et al.*, 2010) ROS levels may be used as an early indicator of future sperm count and quality decline as a result of chronic toxic action of xenobiotics.

The synchronous intensification of lipid peroxidation in rat testis and epididymal suspension of spermatozoids (Table 1) may be the result of *in situ* formation of metabolites of antituberculosis agents. For understanding their exact role in oxidative stress production, germ cells maturation and function, it is important to emphasize that some studies indicated the presence of high-inducible cytochrome P-450 2E1 isoform in male gonads (Healy *et al.*, 1999; Oropeza-Hernandez *et al.*, 2003; Quintans *et al.*, 2005). At least isoniazid and rifampicin may act as cytochrome P-450 2E1 inducers (Madan *et al.*, 2003). CYP 2E1 generates reactive oxygen intermediates, such as superoxide radicals, which in turn could rapidly react with organic molecules generating secondary free radicals and reactive oxygen radical species (Lieber, 1997). Such cascades may alter the reducing milieu of testis and epididymis, producing conditions for sperm oxidative damage. Excessive free radicals generation often involves errors in spermiogenesis and as a result the release of spermatozoa from the germinal epithelium with abnormally high levels of cytoplasmic retention (Sanocka *et al.*, 2004). Lipid peroxidation can profoundly affect sperm quality, including the percentage of motility and specific motility parameters (Bansal *et al.*, 2011).

Glutathione (GSH) is the most abundant non-protein thiol in mammalian cells. Cellular GSH plays a key role in biological processes, including proteins and DNA synthesis and amino acid transport. However, its most important role is the protection of cells against oxidation, including control of male fertility (Luberda, 2005). The sulfhydric group (SH) is a strong nucleophilic group which confers protection against damage by oxidants, electrophilic agents and free radicals. High concentrations of GSH have been observed in rat and mouse testes. A 3-fold increase in the concentration of GSH in rat testis was observed during the onset of spermatogenesis (Donnelly *et al.*, 2000). Isolated hamster spermatocytes and spermatids contained large amounts of reduced GSH, they synthesized GSH and used GSH-dependent defence mechanisms (Donnelly *et al.*, 2000). Changes caused by antituberculosis drugs co-administration in our experiment could be the result of their metabolic transformations *in situ* and the negative effect of metabolites on spermatogenesis processes and structure-functional characteristics of spermatozoids.

Some investigations indicated that apoptosis in cells caused by oxidative stress could be started endogenously by compounds which interact as mediators with receptor systems (Fas/Fas ligands, TNF) (Wang *et al.*, 2005). The intensification of lipid peroxidation as a result of oxidative stress development induces overexpression of the mammalian apoptosis regulator – protooncogen Bcl-2 (Chen *et al.*, 1998). There is also another endogenous way in which the stress signals act as mediators (Wang *et al.*, 2005). The character of DNA fragmentation in human spermatozoa closely correlates with the chemical nature of oxidative base adducts and impaired spermiogenesis incidence (Aitken *et al.*, 2011). Oxidative stress impedes spermatogenesis, resulting in the generation of spermatozoa with poorly remodelled chromatin. These defective cells have a tendency to default to an apoptotic pathway associated with motility loss, caspase activation, phosphatidylserine exteriorization and the activation of free radical generation by mitochondria. The latter induces lipid peroxidation and oxidative DNA damage, which leads to DNA fragmentation and cell death. The physical architecture of spermatozoa prevents any nucleases, activated as a result of this apoptotic process, from gaining access to the nuclear DNA and inducing its fragmentation. Simultaneously, oxidative stress is a key event which starts nonprogrammable cell death (Hakansson *et al.*, 1995).

Differences in DNA fragmentation in experimental and control groups may be caused by activation of different sets of nucleases (Hakansson *et al.*, 1995) and different rates of lipid peroxidation (Aitken *et al.*, 2011). Depending on the quality and quantity of nucleases, the levels of DNA oxidative damage DNA fragmentation results in high or low molecular weight fractions only or in high and lower molecular weight fractions simultaneously (Hakansson *et al.*, 1995; Aitken *et al.*, 2011).

The changes in DNA fragmentation have a more profound character in comparison with changes in testis morphology (unpublished observations). This is in good correspondence with other authors’ data on the possibility of DNA fragmentation disturbances proceeding not only to further apoptotic processes but also to toxic cell death events (Ray *et al.*, 1993). The DNA damage in male germ cells can be accompanied with poor fertilization rates, defective preimplantation embryonic development, high rates of miscarriage and morbidity in the offspring (Aitken *et al.*, 2007). In our experiments postimplantational lethality may have been caused by genotoxic action of substances (Clegg *et al.*, 2001). This assumption could be confirmed by the data of experiments on mice demonstrating weak genotoxicity of pyrazinamide at doses of 125, 250 and 500 mg/kg b.w. (Anitha *et al.* 1994). Moreover, in vitro experiments showed that one isoniazid metabolite – mono acethylhydrazine – increased the number of *Salmonella typhimurium* TA100 and TA1535 revertant mutations and the number of micronuclei in polychromatophylic erythrocytes, which could be evidence of its mutagenic action (Bhide *et al.* 1984). The weak mutagenic effect of isoniazid and its ability to cause liver DNA injury was also demonstrated experimentally (Braun *et al.*, 1984; Yue *et al.*, 2009). Rifampicin genotoxicity investigation showed increased frequency of sister chromatids exchanges in bone marrow cells at doses of 160, 240 and 310 mg/kg b.w. and a number of chromosomal aberrations of spermatocytes at the dose of 80 mg/kg b.w. (Aboul-Ela, 1995).

As to preimplantational lethality, it must be stressed that for exact determination of the involvement of medicines in mutagenic as well as nonmutagenic effects (such as non adequate number of normal and active spermatozoids, disturbances in their transport and penetration into the ovum) additional experiments of mutagenicity and spermatotoxicity of the given drugs must be carried out. At least for ethambutol, it was established that at doses of 25 mg/kg b.w. and 250 mg/kg b.w. this compound caused spermatogenic epithelium disturbances with further blocking of spermatogenesis in rats and cocks (Asole *et al.*, 1976).

Combined administration of therapeutic doses of ethambutol, isoniazid, rifampicin and pyrazinamide to male rats during the whole period of spermatogenesis caused an increase of TBA-active compound contents in rat testis and epididymis, decrease of testis glutathione and protein SH-group contents, profound changes in DNA fragmentation, fatal lowering of male fertilizing capacity and fertility, and increasing levels of pre- and postimplantation embriolethality. The changes in reproductive indices could be the result of direct or mediated action of one or more of the drugs used.
